# Navigating Homogeneous Graph Paths Through Amyloidogenic and Non‐Amyloidogenic Hexapeptides

**DOI:** 10.1002/jcc.70238

**Published:** 2025-10-03

**Authors:** László Keresztes, Evelin Szögi, Bálint Varga, Viktor Farkas, András Perczel, Vince Grolmusz

**Affiliations:** ^1^ PIT Bioinformatics Group Eötvös University Budapest Hungary; ^2^ HUN‐REN‐ELTE Protein Modeling Research Group Eötvös University Budapest Hungary; ^3^ Laboratory of Structural Chemistry and Biology Eötvös University Budapest Hungary; ^4^ Uratim Ltd. Budapest Hungary

**Keywords:** amyloids, hexapeptides, mutational graph, peptides

## Abstract

Hexapeptides are increasingly applied as model systems for studying the amyloidogenic properties of oligo‐ and polypeptides. It is possible to construct 64 million different hexapeptides from the twenty proteinogenic amino acid residues. Today's experimental amyloid databases contain only a fraction of these annotated hexapeptides. For labeling all the possible hexapeptides as “amyloidogenic” or “non‐amyloidogenic” there exist several computational predictors with good accuracy. It may be of interest to define and study a simple graph structure on the 64 million hexapeptides as nodes, when two hexapeptides are connected by an edge if they differ by only a single residue. For example, in this graph, HIKKLM is connected to AIKKLM, or HIKKNM, or HIKKLC, but it is not connected with an edge to VVKKLM or HIKNPM. In the present contribution, we consider our previously published artificial intelligence‐based tool, the Budapest Amyloid Predictor (BAP for short), and demonstrate a spectacular property of this predictor in the graph defined above. We show that for any two hexapeptides predicted to be “amyloidogenic” by the BAP predictor, there exists an easily constructible path of length at most six that passes through neighboring hexapeptides all predicted to be “amyloidogenic” by BAP. For example, the predicted amyloidogenic ILVWIW and FWLCYL hexapeptides can be connected through the length‐6 path ILVWIW‐IWVWIW‐IWVCIW‐IWVCIL‐FWVCIL‐FWLCIL‐FWLCYL in such a way that the neighbors differ in exactly one residue, and all hexapeptides on the path are predicted to be amyloidogenic by BAP. The symmetric statement also holds true for non‐amyloidogenic predicted hexapeptides: For any such pair, there exists a path of length at most six, traversing only predicted non‐amyloidogenic hexapeptides. It is noted that the mentioned property of the Budapest Amyloid Predictor https://pitgroup.org/bap is not proprietary; it is also true for any linear Support Vector Machine (SVM)‐based predictors; therefore, for any future improvements of BAP using the linear SVM prediction technique.

## Graphical abstract

Every two predicted amyloidogenic hexapeptides can be connected by a path of length at most six, by changing only one amino acid in each step, and going through predicted amyloidogenic hexapeptides only.

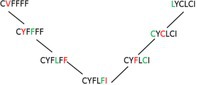

## 1 Introduction

Amyloids are misfolded proteins with a well‐defined parallel and/or antiparallel repeating β‐sheet structure [1, 2]. Numerous globular proteins can turn into amyloids in certain physical or chemical environments [2]. While amyloids are most frequently mentioned in the context of human diseases [3], they can also be functional building blocks in healthy human tissues [4] or can serve as prospective anti‐viral agents [5].

In the last decade, hexapeptides have become a popular class of molecules for modeling and studying the protein amyloid formation: These short peptides are simple enough to be studied in a variety of in vitro and in silico systems, yet complex enough to show characteristic amyloid formation changes in numerous studies. Because of their applicability as model systems, experimental data have been collected on hundreds of hexapeptides in relation to their amyloidogenic properties. The creators of the Waltz database [6, 7] published 1415 hexapeptides, of which 514 were experimentally labeled as “amyloidogenic” and 901 as “non‐amyloidogenic”.

By applying the labeled molecules from the Waltz database for training an artificial intelligence tool, our research group has prepared a support vector machine [8] (SVM)‐based tool for amyloidogenicity‐prediction for hexapeptides [9]. Our tool, called the Budapest Amyloid Predictor (BAP), is publicly available at https://pitgroup.org/bap. We have shown in [9] that the accuracy of the BAP predictor is 84% (and the further quality measures are TPR=0.75, TNR=0.9, PPV=0.8, NPV=0.86; i.e., true positive ratio, true negative ratio, positive predictive value, negative predictive value, resp.).

A recent review of published amyloid‐predictors [10] lists, among others, Zyggregator [11], AGGRESCAN [12], netCSSP [13], APPNN [14]. Our BAP has the same or better accuracy as the predictors listed in [10], as it was shown in [9].

The BAP predictor is based on a linear Support Vector Machine (SVM) [8]. SVM‐based predictors have a much more transparent structure than other artificial intelligence predictors, and this transparency leads to very strong applications. Generally, it is difficult to explain the intrinsic “reason” by which a deep neural network predictor makes a decision or to describe those attributes of the input that lead to a given classification by the network.

The transparent structure of the SVM predictor BAP [9] was exploited in our work [15], where we have identified patterns, describing amyloid‐forming hexapeptides very succinctly. For example, we have shown that for any substitution with the 20 proteogenic amino acids for positions denoted by x, all the patterns CxFLWx, FxFLFx, or xxIVIV are predicted amyloidogenic, and all the patterns PxDxxx, xxKxEx, and xxPQxx are predicted non‐amyloidogenic. We note that any pattern with two x's describes $20^{2}=400$ hexapeptides, and patterns with four x's describe $20^{4}=160,000$ hexapeptides. In [15] we have described all such patterns, and also amyloidogenic patterns with restricted choices for the positions of x, where the residues were allowed to be selected from polar, non‐polar, or hydrophobic subsets of the 20 proteogenic amino acids.

We note that the transparent structure of the Support Vector Machines made it possible to identify different patterns in [15, 16, 17].

In the present contribution, we exploit further the transparent structure of the predictor BAP https://pitgroup.org/bap. Suppose we want to find a path from a hexapeptide x to another hexapeptide $x^{\prime }$ through different hexapeptides, such that in each step, we can move from one hexapeptide to another with exactly one different residue position.

Note on the terminology: When a sequence of reactions is studied, the “pathway” term is used generally. We apply here the graph‐theoretical, more abstract “path” term, since we work in the present contribution on a graph.

For example, we want to find a path from hexapeptide ILVWIW to hexapeptide FWLCYL, through six‐tuples, differing in exactly one residue. An obvious path is generated by changing the amino acids one by one from left to right, starting from ILVWIW and finishing at FWLCYL as follows:

$$\begin{array}{ll} & \text{ILVWIW‐FLVWIW‐FWVWIW‐FWLWIW}\;\\ & \;\text{‐FWLCIW‐FWLCYW‐FWLCYL}\; \end{array}$$

These paths from one‐by‐one residue‐exchanges can be of interest in peptide synthesis design or following a sequence of point mutations of peptides or protein sequences and measuring or modeling the change of their subsequent chemical or biological properties when only one residue is altered in one step.

Analyzing the effects of subsequent point mutations was done in the literature in the past decades. In [18], three different biologically active peptides were transformed into each other by subsequent single amino acid substitutions, and the intermediaries were analyzed for activity. The authors of [18] called the paths formed from the subsequent point‐mutated peptides “evolutionary transition pathways”.

Paths of one‐by‐one residue exchanges can be interesting, which connect two predicted amyloidogenic hexapeptides and go through amyloidogenic hexapeptides only.

Similarly, we may want to design paths between the non‐amyloidogenic hexapeptides A and B, along which only one residue is changed in each step, and which go through non‐amyloidogenic intermediaries only.

In the present contribution, we show the following results for the BAP predictor:

- All predicted amyloidogenic pairs of hexapeptides, x and $x^{\prime }$, can be connected by one‐by‐one exchanged residue‐paths of length at most six, such that the whole path contains only predicted amyloidogenic intermediaries. Moreover, the path can be computed easily.
- All predicted non‐amyloidogenic pairs of hexapeptides, x and $x^{\prime }$, can be connected by one‐by‐one exchanged residue‐paths of length at most six, such that the whole path contains only predicted non‐amyloidogenic intermediaries. Moreover, the path can be computed easily.

We also show that the same results hold for other linear‐SVM‐based predictors, and not only for our BAP predictor described in [9].

We remark that in the case of non‐SVM‐based predictors, it may happen that two predicted amyloidogenic sequences cannot be connected by entirely amyloidogenic paths of *any* length, and the same holds for the non‐amyloidogenic case, too. For example, in non‐SVM‐based predictors, it may happen that all the neighbors of an amyloidogenic peptide A are predicted to be non‐amyloidogenic; consequently, A cannot be connected by an entirely amyloidogenic path to any other amyloidogenic peptide.

We also remark that we do not state anything on paths connecting amyloidogenic hexapeptides with non‐amyloidogenic ones.

## 2 Methods

Here, we first formalize our problem setting and solution, and then we will make some remarks on possible generalizations.

All our definitions and methods or algorithms will be specified for hexapeptide sequences, but they are easily generalizable for shorter or longer amino acid sequences of a given length.

First, we define the mutation‐graph M on the hexapeptide sequences:

Definition 1 The vertices of the mutation‐graph M are the $20^{6}=64$ million hexapeptides formed from the 20 proteogenic amino acids. The vertices are referred to using their length‐6 amino acid sequences with the one‐letter codes. Two vertices of M are connected by an edge if they differ in exactly one amino acid in the same position.

Example 1 Node ILVWIW is connected by an edge to ALVWIW, or to IAVWIW, or to ILVWID, but not to IDDWIW.

We note that paths in this graph M were called “evolutionary transition pathways” in [18]. We simply call them “paths” in M. The length of a path is the number of edges in it.

It is easy to see that in each position, we can make 19 different substitutions (the original amino acid can be substituted by any of the remaining 20−1=19 proteogenic amino acids), and since we have six positions, every vertex is connected to 6×19=114 other nodes, which represent exactly 114 hexapeptides.

Next, we partition the vertices of M into two classes: Amyloidogenic and non‐amyloidogenic. That is, each vertex is an element of one and only one of those classes. The partitioning is done by the Budapest Amyloid Predictor, described in detail in [9].

### 2.1 The Budapest Amyloid Predictor and the Amyloid Effect Matrix

Here, we succinctly describe the BAP predictor with details needed to prove our statement and to show our method for finding the paths, leading entirely within one of the two partition classes. The details of the construction of the Budapest Amyloid Predictor, the evaluation of its correctness, and the comparison with other predictors were described in detail in [9].

The BAP predictor uses a linear Support Vector Machine (SVM) [8] for decisions. A linear SVM computes the sign of the value

(1) $$\overset{n}{\underset{i=1}{\sum }}w_{i}z_{i}+b$$

and it makes a decision based on this sign. Here, the coefficients $w_{1},w_{2},\text{…},w_{n}$ and b are real numbers computed from the training data, and $z_{1},z_{2},\text{…},z_{n}$ represent the input values. For example, if for a given input $z=(z_{1},z_{2},\text{…},z_{n})$ the value of (1) is non‐negative, the SVM outputs “yes” otherwise “no”.

The Budapest Amyloid Predictor [9] (available at https://pitgroup.org/bap) applied the Waltz dataset [6, 7] for training and testing an SVM, where each of the 20 proteogenic amino acids was represented as a (highly redundant) length‐553 vector Z, corresponding to 553 properties of AAindex [19]. Therefore, a hexapeptide was represented by six concatenated Z vectors; their combined length is 6×553=3318=n.

With ℓ=553, Equation (1) can be written as

(2) $$\overset{6\ell }{\underset{i=1}{\sum }}w_{i}z_{i}+b=\overset{6}{\underset{j=1}{\sum }}\;\;\overset{j\ell }{\underset{i=(j-1)\ell +1}{\sum }}w_{i}z_{i}+b$$

If the value of (2) is negative (i.e., its sign is −1), the hexapeptide is predicted to be non‐amyloidogenic; if it is positive or 0, it (its sign is 1 or 0) is predicted to be amyloidogenic.

Here, index j refers to amino acid j in the hexapeptide for j=1,2,…,6. Since the $\ell =553\;z_{i}^{\prime }$s are determined by the jth amino acid of the hexapeptide, and this way, all the possible 6×20=120 second sums in Equation (2) (for six positions and 20 amino acids) can be pre‐computed.

Table 1 lists these pre‐computed values: The six values of j correspond to the columns, the amino acids to the rows. In other words, Table 1, which is called the “Amyloid Effect Matrix” in [9], describes the position‐dependent contributions of amino acids to the value of (2).

**TABLE 1:** The Amyloid Effect Matrix [9]: The pre‐computed values from Equation (2) are listed in the rows corresponding to the amino acids. The columns correspond to the positions in the hexapeptide.

	1	2	3	4	5	6
A	−0.26	−0.32	−0.27	−0.14	−0.43	−0.22
R	−0.45	−0.41	−0.46	−0.33	−0.52	−0.35
N	−0.40	−0.34	−0.49	−0.27	−0.46	−0.30
D	−0.49	−0.43	−0.56	−0.41	−0.56	−0.36
C	−0.09	−0.21	0.03	−0.05	−0.17	−0.05
Q	−0.37	−0.30	−0.36	−0.34	−0.48	−0.32
E	−0.51	−0.41	−0.43	−0.30	−0.61	−0.39
G	−0.23	−0.37	−0.46	−0.37	−0.30	−0.33
H	−0.32	−0.26	−0.26	−0.30	−0.35	−0.25
I	−0.06	−0.08	0.26	0.09	−0.06	−0.07
L	−0.10	−0.18	0.02	0.04	−0.22	−0.13
K	−0.39	−0.45	−0.51	−0.35	−0.59	−0.32
M	−0.17	−0.25	−0.02	−0.10	−0.19	−0.18
F	−0.13	−0.11	0.05	−0.03	−0.13	−0.11
P	−0.56	−0.38	−0.56	−0.51	−0.42	−0.45
S	−0.37	−0.35	−0.41	−0.30	−0.48	−0.23
T	−0.34	−0.33	−0.28	−0.23	−0.40	−0.23
W	−0.17	−0.17	−0.09	−0.06	−0.12	−0.16
Y	−0.23	−0.11	−0.13	−0.06	−0.18	−0.15
V	−0.05	−0.14	0.19	0.14	−0.19	0.01

Table 1 facilitates the easy “by hand” computation of sum (2) and making a decision on its amyloidogenicity. For example, if we want to make a prediction on YVSTSY, then we need to take the value from column 1, corresponding to Y (i.e., −0.23), and from column 2, corresponding to V (−0.14), from column 3, corresponding to S (−0.41), from column 4 in row of T (−0.23), from column 5 in the row of S (−0.48), and from column 6 corresponding to Y (−0.15), add them up, and add b=1.083 to the sum:$\begin{array}{c} -0.23-0.14-0.41-0.23-0.48-0.15+1.083=-0.557; \end{array}$ therefore, YVSTSY is predicted to be non‐amyloidogenic.

One can simply order the amino acids in each position of the hexapeptides according to their contribution to the sum (2), as in Table 2.

**TABLE 2:** The position‐specific amyloidogenicity order of the amino acids, decreasing from left to right, [9].

	1	2	3	4	5	6	7	8	9	10	11	12	13	14	15	16	17	18	19	20
1	V	I	C	L	F	M	W	G	Y	A	H	T	S	Q	K	N	R	D	E	P
2	I	F	Y	V	W	L	C	M	H	Q	A	T	N	S	G	P	R	E	D	K
3	I	V	F	C	L	M	W	Y	H	A	T	Q	S	E	R	G	N	K	D	P
4	C	I	L	F	C	W	Y	M	A	T	N	H	E	S	R	Q	K	G	D	P
5	I	W	F	C	Y	M	V	L	G	H	T	P	A	N	Q	S	R	D	K	E
6	V	C	I	F	L	Y	W	M	A	T	S	H	N	Q	K	G	R	D	E	P

Table 2 has some very practical applications for amyloidogenesis prediction. If we have one predicted amyloidogenic hexapeptide x, then we can easily make numerous other predicted amyloidogenic hexapeptides from x, simply by replacing any amino acid in a given position by one, which is situated left to the original one in its row in Table 2. More exactly, if hexapeptide x is predicted to be amyloidogenic, and its 3rd amino acid is Y, then Y can be exchanged to either of I, V, F, C, L, M, or W, the resulting hexapeptide $x^{\prime }$ will always be predicted to be amyloidogenic. This is true since Table 2 contains the orderings of the amino acids in each position according to their contribution in Table 1, and if we exchange Y to anything from its left in row 3 of Table 2, then we increase the value of the sum of (2), relative to that of x. Since the value of the sum in the case of x was positive, its increased value will also be positive, that is, the decision of the SVM will be “amyloidogenic”.

Similarly, in the case of a hexapeptide n, predicted to be non‐amyloidogenic, if we exchange any amino acids located to the right of the original in Table 2, then the new prediction will also be non‐amyloidogenic. For example, if the 4th amino acid of n is E, and we exchange E to any of the S, R, Q, K, G, D, P, then the value of sum (2) will be decreased, and, consequently, the prediction will remain non‐amyloidogenic.

The description of the path constructions will be more convenient by introducing two simple operators, for i=1,2,3,4,5,6, and for any two (not necessarily distinct) amino acids X and $X^{\prime }$:

$${\mathrm{MAX}}_{i}(X,X^{\prime })=\;\text{In row i of Table 2 the leftmost one of}\;(X,X^{\prime })$$

$${\mathrm{MIN}}_{i}(X,X^{\prime })=\;\text{In row i of Table 2 the rightmost one of}\;(X,X^{\prime }).$$

If $X=X^{\prime }$ in any of the two operators, then the output value is $X=X^{\prime }$.

The MAX and MIN terms refer to the amyloidogenicity of the amino acids in position i.

Let us call the value (2) of a hexapeptide x its amyloidogenicity value, and let us denote it by A(x). If A(x)≥0, then x is predicted to be amyloidogenic, otherwise non‐amyloidogenic.

## 3 Results

Here, we show how to connect any two hexapeptides of the same amyloidogenicity prediction with a path of length at most six, going on the same class as their endpoints in graph M, the mutation graph.

### 3.1 Constructing Paths Through the Amyloidogenic Hexapeptides

Suppose we have two hexapeptides, $x=(X_{1},X_{2},X_{3},X_{4},X_{5},X_{6})$ and $x^{\prime }=(X_{1}^{\prime },X_{2}^{\prime },X_{3}^{\prime },X_{4}^{\prime },X_{5}^{\prime },X_{6}^{\prime })$, both predicted to be amyloidogenic by BAP. For simplicity, we will call the $X_{i}$ amino acids “coordinates” of x.

Now we show that there exists an easily constructible path of length at most six in the graph M, such that all vertices of the path are predicted amyloidogenic.

Case 1 (the easy case) Suppose that $x^{\prime }$ is “coordinate‐wise more amyloidogenic” than x in the following sense: For all i=1,2,3,4,5,6, either $X_{i}=X_{i}^{\prime }$, or $X_{i}^{\prime }$ is situated left from $X_{i}$ in row i of Table 2; that is, $X_{i}$ is less amyloidogenic in position i than $X_{i}^{\prime }$. Then, if we change $X_{i}$ to $X_{i}^{\prime }$ in position i, for i=1,2,3,4,5,6, then we go through a path from x to $x^{\prime }$ in graph M such that the value of A on the nodes, describing the amyloidogenicity, will be monotone increasing. Therefore, all nodes on the path will be predicted to be amyloidogenic. Note that the length of this path is at most six: When the same amino acid appears in the same coordinates, no change is needed; when every coordinate is different, then six changes are needed.

Formally:

$$A(x)=A(X_{1},X_{2},X_{3},X_{4},X_{5},X_{6})\leq A(X_{1}^{\prime },X_{2},X_{3},X_{4},X_{5},X_{6})\leq$$

$$\begin{array}{ll} & \leq A(X_{1}^{\prime },X_{2}^{\prime },X_{3},X_{4},X_{5},X_{6})\;\\ & \leq \text{⋯}\leq A(X_{1}^{\prime },X_{2}^{\prime },X_{3}^{\prime },X_{4}^{\prime },X_{5}^{\prime },X_{6}^{\prime })=A(x^{\prime })\; \end{array}$$

Case 2 (the general case) When the assumptions of Case 1 are not satisfied, we reduce the problem to two applications of path‐finding in Case 1.

Our strategy is as follows:

Step I: First, we connect node $x=(X_{1},X_{2},X_{3},X_{4},X_{5},X_{6})$ to node
$$x_{\mathrm{MAX}}=({\mathrm{MAX}}_{1}(X_{1},X_{1}^{\prime }),{\mathrm{MAX}}_{2}(X_{2},X_{2}^{\prime }),{\mathrm{MAX}}_{3}(X_{3},X_{3}^{\prime }),$$
$${\mathrm{MAX}}_{4}(X_{4},X_{4}^{\prime }),{\mathrm{MAX}}_{5}(X_{5},X_{5}^{\prime }),{\mathrm{MAX}}_{6}(X_{6},X_{6}^{\prime })),$$
  exactly as in Case 1, since they satisfy the assumptions.
Step II: Second, we connect $x^{\prime }$ to $x_{\mathrm{MAX}}$, as in Case 1, since they satisfy the assumptions.

Now, we detail that in both Steps I and II, the requirements of Case 1 are satisfied. Since both x and $x^{\prime }$ are amyloidogenic, and since both $A(x)\leq A(x_{\mathrm{MAX}})$ and $A(x^{\prime })\leq A(x_{\mathrm{MAX}})$ hold, $x_{\mathrm{MAX}}$ is also “coordinate‐wise more amyloidogenic” than both x and $x^{\prime }$.

In other words, because of the definition of the ${\mathrm{MAX}}_{i}$ operators, the coordinates of $x_{\mathrm{MAX}}$ are left in Table 2 from the coordinates x and $x^{\prime }$ in each row. Therefore, in Step I, the procedure of Case 1 can be applied for connecting x to $x_{\mathrm{MAX}}$; and in Step II, the procedure of Case 1 can be applied to connect $x^{\prime }$ to $x_{\mathrm{MAX}}$. Since the paths are undirected, we take the path x to $x_{\mathrm{MAX}}$ and further to $x^{\prime }$.

Now we show that the combined length of the path from x to $x_{\mathrm{MAX}}$, and from $x_{\mathrm{MAX}}$ to $x^{\prime }$ is at most six: It is easy to verify that for all i, ${\mathrm{MAX}}_{i}(X_{i},X_{i}^{\prime })$ is either equal to $X_{i}$ or $X_{i}^{\prime }$, so if an exchange is needed in Step I in coordinate i, then no change is needed in Step II in coordinate i, and a symmetric remark is also true for Steps II and I.

Example 2 Let us connect hexapeptides x=CVFFFF to $x^{\prime }=$LYCLCI by a predicted amyloidogenic path. Both x and $x^{\prime }$ are predicted to be amyloidogenic. Case 1 cannot be applied (one can see it easily from Table 2), so we need $x_{\mathrm{MAX}}=\text{CYFLFI}$. So, we first connect x to $x_{\mathrm{MAX}}$:

$$\begin{array}{c} CVFFFF-CYFFFF-CYFLFF-CYFLFI \end{array}$$

Then $x^{\prime }$ to $x_{\mathrm{MAX}}$:

$$\begin{array}{c} LYCLCI-CYCLCI-CYFLCI-CYFLFI \end{array}$$

The full path is:

$$\begin{array}{c} \begin{array}{ll} & CVFFFF-CYFFFF-CYFLFF-CYFLFI\;\\ & \;-CYFLCI-CYCLCI-LYCLCI\; \end{array} \end{array}$$

### 3.2 Constructing Paths Through the Non‐Amyloidogenic Hexapeptides

The proof of this case is the repetition of the construction above, with the obvious changes. For completeness, we give here the proof.

Suppose we have two hexapeptides, $x=(X_{1},X_{2},X_{3},X_{4},X_{5},X_{6})$ and $x^{\prime }=(X_{1}^{\prime },X_{2}^{\prime },X_{3}^{\prime },X_{4}^{\prime },X_{5}^{\prime },X_{6}^{\prime })$, both predicted to be non‐amyloidogenic by BAP.

We show that there exists an easily constructible path of length at most six in the graph M, such that all vertices of the path are predicted non‐amyloidogenic.

Case 1 (the easy case) Suppose that for all i=1,2,3,4,5,6, either $X_{i}=X_{i}^{\prime }$, or $X_{i}^{\prime }$ is situated right from $X_{i}$ in row i of Table 2; that is, $X_{i}^{\prime }$ is less amyloidogenic in position i than $X_{i}$. Then, if we change $X_{i}$ to $X_{i}^{\prime }$ in position i, for i=1,2,3,4,5,6, we go through a path in graph M from x to $x^{\prime }$ such that the value of A on the nodes, describing the amyloidogenicity, will be monotone decreasing. Note that the length of this path is at most six: When the same amino acid appears in the same coordinates, no change is needed; when every coordinate is different, then six changes are needed.

More formally:

$$A(x)=A(X_{1},X_{2},X_{3},X_{4},X_{5},X_{6})\geq A(X_{1}^{\prime },X_{2},X_{3},X_{4},X_{5},X_{6})\geq$$

$$\begin{array}{ll} & \geq A(X_{1}^{\prime },X_{2}^{\prime },X_{3},X_{4},X_{5},X_{6})\;\\ & \geq \text{…}\geq A(X_{1}^{\prime },X_{2}^{\prime },X_{3}^{\prime },X_{4}^{\prime },X_{5}^{\prime },X_{6}^{\prime })=A(x^{\prime })\; \end{array}$$

Case 2 (the general case) When the assumptions of Case 1 are not satisfied, we reduce the problem to two applications of path‐finding in Case 1.

Our strategy is as follows:

Step I: First, we connect node $x=(X_{1},X_{2},X_{3},X_{4},X_{5},X_{6})$ to node
$$x_{\mathrm{MIN}}=({\mathrm{MIN}}_{1}(X_{1},X_{1}^{\prime }),{\mathrm{MIN}}_{2}(X_{2},X_{2}^{\prime }),{\mathrm{MIN}}_{3}(X_{3},X_{3}^{\prime }),$$
$${\mathrm{MIN}}_{4}(X_{4},X_{4}^{\prime }),{\mathrm{MIN}}_{5}(X_{5},X_{5}^{\prime }),{\mathrm{MIN}}_{6}(X_{6},X_{6}^{\prime })),$$
  exactly as in Case 1, since they satisfy the assumptions.
Step II: Second, we connect $x^{\prime }$ to $x_{\mathrm{MIN}}$, as in Case 1, since they satisfy the assumptions.

Now, we detail that in both Steps I and II, the requirements of Case 1 are satisfied. Since both x and $x^{\prime }$ are amyloidogenic, and since both $A(x)\geq A(x_{\mathrm{MIN}})$ and $A(x^{\prime })\geq A(x_{\mathrm{MIN}})$ hold, $x_{\mathrm{MIN}}$ is also coordinate‐wise less amyloidogenic than both x and $x^{\prime }$.

In other words, because of the definition of the ${\mathrm{MIN}}_{i}$ operators, the coordinates of $x_{\mathrm{MIN}}$ are right in Table 2 from the coordinates x and $x^{\prime }$ in each row. Therefore, in Step I, the procedure of Case 1 can be applied for connecting x to $x_{\mathrm{MIN}}$; and in Step II, the procedure of Case 1 can be applied to connect $x^{\prime }$ to $x_{\mathrm{MIN}}$. Since the paths are undirected, we take the path x to $x_{\mathrm{MIN}}$ and further to $x^{\prime }$.

Now we show that the combined length of the path from x to $x_{\mathrm{MIN}}$, and from $x_{\mathrm{MIN}}$ to $x^{\prime }$ is at most six: It is easy to verify that for all i, ${\mathrm{MIN}}_{i}(X_{i},X_{i}^{\prime })$ is either equal to $X_{i}$ or $X_{i}^{\prime }$, so if an exchange is needed in Step I in coordinate i, then no change is needed in Step II in coordinate i, and a symmetric remark is also true for Steps II and I.

## 4 Conclusions

We have shown that the linear SVM predictors for peptides have a very transparent structure that can be used to design mutational pathways within the predicted classes. More specifically, we have used the Budapest Amyloid Predictor [9] to partition 64 million possible hexapeptides into two classes: Predicted amyloidogenic and predicted non‐amyloidogenic, and we have shown that any two members of each class can be connected by a mutation pathway of length at most six that lies entirely within the same class, that is, amyloidogenic or non‐amyloidogenic. For the construction, we used Table 2, defined by the Budapest Amyloid Predictor. The same result can be obtained using any other updated version of Table 2, so our results here are not specific to the Budapest Amyloid Predictor.

## Author Contributions

**László Keresztes**, **Evelin Szögi**, **András Perczel**, **Viktor Farkas** and **Vince Grolmusz:** have initiated the study and evaluated results; **László Keresztes** and **Evelin Szögi:** constructed the SVM for the prediction; **Bálint Varga:** has constructed the web server; **Vince Grolmusz:** has overseen the work and written the first version of the paper. All authors have reviewed the article. **András Perczel**, **Viktor Farkas** and **Vince Grolmusz:** secured funding.

## Conflicts of Interest

The authors declare no conflicts of interest.

## Data Availability

All data are included in the text.

## References

[1] D. Horvath , D. Menyhard , and A. Perczel , “Protein Aggregation in a Nutshell: The Splendid Molecular Architecture of the Dreaded Amyloid Fibrils,” Current Protein and Peptide Science 20, no. 11 (2019): 1077–1088. 31553291 10.2174/1389203720666190925102832

[2] N. Taricska , D. Horvath , D. K. Menyhard , et al., “The Route From the Folded to the Amyloid State: Exploring the Potential Energy Surface of a Drug‐Like Miniprotein,” Chemistry A European Journal 26 (2019): 1968–1978, 10.1002/chem.201903826. 31647140 PMC7028080

[3] C. Soto , L. Estrada , and J. Castilla , “Amyloids, Prions and the Inherent Infectious Nature of Misfolded Protein Aggregates,” Trends in Biochemical Sciences 31, no. 3 (2006): 150–155. 16473510 10.1016/j.tibs.2006.01.002

[4] S. K. Maji , M. H. Perrin , M. R. Sawaya , et al., “Functional Amyloids as Natural Storage of Peptide Hormones in Pituitary Secretory Granules,” Science 325, no. 5938 (2009): 328–332. 19541956 10.1126/science.1173155 PMC2865899

[5] E. Michiels , K. Roose , R. Gallardo , et al., “Reverse Engineering Synthetic Antiviral Amyloids,” Nature Communications 11, no. 1 (2020): 2832, 10.1038/s41467-020-16721-8. PMC7275043 32504029

[6] J. Beerten , J. Van Durme , R. Gallardo , et al., “WALTZ‐DB: A Benchmark Database of Amyloidogenic Hexapeptides,” Bioinformatics (Oxford, England) 31 (2015): 1698–1700, 10.1093/bioinformatics/btv027. 25600945

[7] N. Louros , K. Konstantoulea , M. De Vleeschouwer , M. Ramakers , J. Schymkowitz , and F. Rousseau , “WALTZ‐DB 2.0: An Updated Database Containing Structural Information of Experimentally Determined Amyloid‐Forming Peptides,” Nucleic Acids Research 48 (2020): D389–D393, 10.1093/nar/gkz758. 31504823 PMC6943037

[8] C. Cortes and V. Vapnik , “Support‐Vector Networks,” Machine Learning 20, no. 3 (1995): 273–297.

[9] L. Keresztes , E. Szögi , B. Varga , V. Farkas , A. Perczel , and V. Grolmusz , “The Budapest Amyloid Predictor and Its Applications,” Biomolecules 11, no. 4 (2021): 500, 10.3390/biom11040500. 33810341 PMC8067080

[10] S. Jaime , P. Jordi , P. Irantzu , I. Valentin , and V. Salvador , “Computational Prediction of Protein Aggregation: Advances in Proteomics, Conformation‐Specific Algorithms and Biotechnological Applications,” Computational and Structural Biotechnology Journal 18 (2020): 1403–1413, 10.1016/j.csbj.2020.05.026. 32637039 PMC7322485

[11] G. G. Tartaglia and M. Vendruscolo , “The Zyggregator Method for Predicting Protein Aggregation Propensities,” Chemical Society Reviews 37 (2008): 1395–1401, 10.1039/b706784b. 18568165

[12] O. Conchillo‐Solé , N. S. de Groot , F. X. Avilés , J. Vendrell , X. Daura , and S. Ventura , “Aggrescan: A Server for the Prediction and Evaluation of Hot Spots of Aggregation in Polypeptides,” BMC Bioinformatics 8 (2007): 65, 10.1186/1471-2105-8-65. 17324296 PMC1828741

[13] C. Kim , J. Choi , S. J. Lee , W. J. Welsh , and S. Yoon , “Netcssp: Web Application for Predicting Chameleon Sequences and Amyloid Fibril Formation,” Nucleic Acids Research 37 (2009): W469–W473, 10.1093/nar/gkp351. 19468045 PMC2703942

[14] F. Carlos , R. D. Sarah , Q. Alexandre , and A. P. David , “Prediction of Peptide and Protein Propensity for Amyloid Formation,” PLoS One 10 (2015): e0134679, 10.1371/journal.pone.0134679. 26241652 PMC4524629

[15] L. Keresztes , E. Szogi , B. Varga , V. Farkas , A. Perczel , and V. Grolmusz , “Succinct Amyloid and Non‐Amyloid Patterns in Hexapeptides,” ACS Omega 7, no. 40 (2022): 35532–35537, 10.1021/acsomega.2c02513. 36249386 PMC9558248

[16] L. Keresztes , E. Szogi , B. Varga , and V. Grolmusz , “Identifying Super‐Feminine, Super‐Masculine and Sex‐Defining Connections in the Human Braingraph,” Cognitive Neurodynamics 15, no. 6 (2021): 949–959, 10.1007/s11571-021-09687-w. 34786030 PMC8572280

[17] L. Keresztes , E. Szogi , B. Varga , and V. Grolmusz , “Discovering Sex and Age Implicator Edges in the Human Connectome,” Neuroscience Letters 791 (2022): 136913, 10.1016/j.neulet.2022.136913. 36272557

[18] U. Hoffmüller , T. Knaute , M. Hahn , W. Höhne , J. Schneider‐Mergener , and A. Kramer , “Evolutionary Transition Pathways for Changing Peptide Ligand Specificity and Structure,” EMBO Journal 19, no. 18 (2000): 4866–4874. 10990450 10.1093/emboj/19.18.4866 PMC314224

[19] S. Kawashima , P. Pokarowski , M. Pokarowska , A. Kolinski , T. Katayama , and M. Kanehisa , “Aaindex: Amino Acid Index Database, Progress Report 2008,” Nucleic Acids Research 36 (2008): D202–D205, 10.1093/nar/gkm998. 17998252 PMC2238890

